# Relationships between Eye Movements during Sentence Reading Comprehension, Word Spelling and Reading, and DTI and fmri Connectivity In Students with and without Dysgraphia or Dyslexia

**DOI:** 10.15761/JSIN.1000150

**Published:** 2017-01-18

**Authors:** Kevin Yagle, Todd Richards, Katie Askren, Zoe Mestre, Scott Beers, Robert Abbott, William Nagy, Peter Boord, Virginia Berninger

**Affiliations:** 1 Integrated Brain Imaging Center, Department of Radiology, University of Washington, Seattle, USA; 2 School of Education, Seattle Pacific University, Seattle, USA; 3 Department of Educational Psychology, University of Washington, Seattle, USA

**Keywords:** brain bases of eye movements, eye movement fixations, eye movement saccades, sentence reading comprehension, typical reading and writing, dysgraphia, dyslexia

## Abstract

While eye movements were recorded and brains scanned, 29 children with and without specific learning disabilities (SLDs) decided if sentences they read (half with only correctly spelled words and half with homonym foils) were meaningful. Significant main effects were found for diagnostic groups (non-SLD control, dysgraphia control, and dyslexia) in total fixation (dwell) time, total number of fixations, and total regressions in during saccades; the dyslexia group had longer and more fixations and made more regressions in during saccades than either control group. The dyslexia group also differed from both control groups in (a) fractional anisotropy in left optic radiation and (b) silent word reading fluency on a task in which surrounding letters can be distracting, consistent with Rayner's selective attention dyslexia model. Different profiles for non-SLD control, dysgraphia, and dyslexia groups were identified in correlations between total fixation time, total number of fixations, regressions in during saccades, magnitude of gray matter connectivity during the fMRI sentence reading comprehension from left occipital temporal cortex seed with right BA44 and from left inferior frontal gyrus with right inferior frontoccipital fasciculus, and normed word-specific spelling and silent word reading fluency measures. The dysgraphia group was more likely than the non-SLD control or dyslexia groups to show negative correlations between eye movement outcomes and sentences containing incorrect homonym foils. Findings are discussed in reference to a systems approach in future sentence reading comprehension research that integrates eye movement, brain, and literacy measures.

## Introduction

Psychological science has established that eyes are constantly in motion, even though humans are not consciously aware of this motion [1-3]. When individuals read, their eyes make rapid ballistic eye movements (saccades) followed by moments of relative stability (fixations). During fixations, initial processing of letters and written words begins [3], based on information in stimuli received from the retina's fovea, which is sensitive to visual detail, in contrast to the retina's periphery, which detects contrasts between darkness and brightness. Saccades (forward progressions and backward regressions) alternate with fixations, but the majority of time is devoted to the fixation themselves rather than the forward movements to new fixations or backward regressions to prior fixations or skipped words; and typically more saccades are forward from fixated words than backwards to a prior fixation or skipped word [1,2].

However, not all saccades may be the same. Learning to self-regulate saccades, even if at a subconscious level, involves making “strategic jumps” to upcoming words, correcting if a saccade goes too far, and making regressions when necessary. For example, a reader may have trouble regulating serial forward movement one word at a time [4] and so fixates beyond the target word and has to return to it through a *regression into it*. Alternatively, the regression may reflect self-monitoring during the reading process and indicate that the reader is trying to integrate the currently fixated target with accumulating words in sentence syntax and returns through a *regression out* of the currently fixated word to previous fixation to verify what a prior word or group of words was.

Over four decades of eye movement research have shed light on the mind's mental processes during reading [1-3,5,6]. Presumably, where the mind's eye is “looking” during fixation is a window on the mind's focus of internal attention; thus, eye movement studies are a valuable research tool for studying mental attention during reading [3]. However, two key principles should be kept in mind in interpreting eye movement data. First, the locus of fixation is not necessarily the locus of attention; visual processing can occur on a word or letter string without direct fixation to it [5]. Second, both bottom-up attention processes influenced by properties of incoming stimuli and top-down attention processes involved in self-regulation of mental activity [7] may play important roles in both eye movements and orchestration of the full reading process. Furthermore, the nature of eye movements and the self-regulation of mental attention may change over the course of development in general and reading in particular. Whereas some eye movement research has been specific to reading only in children or only in adults, other cross sectional eye movement research has focused on eye movements in both children and adults.

However, throughout development eye movements are influenced by word length, meaningfulness of the word in the context of other words [8-10], and integration of words across saccades into multi-word units [1-3]. According to the E-Z Reader serial attention model of eye movements in reading [4], attention is directed serially to one word at a time; and completion of initial processing of a word initiates saccadic programming to move eyes to the next word at the n + 1 location. If a word is not accessed and integrated within an unfolding sentence, then an inter-word or intra-word regression occurs as eyes and attention seek sources of the inter-word integration difficulty. A competing gradient (Swift) model posits that more than one word can be processed lexically at a given time [2,11]. Despite such conceptual differences between models, a consensus has emerged that eye movements are not just an oculomotor skill, but also draw on linguistic processing at the word-level and at the sentence syntax-level, whether in sequence or in parallel or a combination of serial and parallel. Top-down influences on sentence reading comprehension may, therefore, draw not only on attentional influences but also executive coordination of different levels of language.

Yet another influence on eye movements during reading is individual differences among readers. Most eye movement research has focused on typically developing readers, but some studies have focused on typically developing children and youth with reading disabilities such as dyslexia. Rayner's research showed that dyslexia is not caused by faulty eye movements; rather less efficient eye movements are a consequence of dyslexia [1]. For example, Rayner and colleagues introduced the concept of selective attention dyslexia, in which letters from parafoveal vision interfere with processing of a currently fixated word [12]. More recently Jainta and Kappoula studied several oculomotor parameters essential for efficient reading, including control of saccade movements [13]. Jainta and Kappoula's findings showed that those with dyslexia had (a) more fixations and more regressions than those who did not have dyslexia, consistent with prior findings of Pavlidis [14] and Kirby and colleagues [15], and (b) slightly longer fixation durations and a tendency toward larger saccade amplitudes. See Zuber and Stark for an overview of research on relationships of saccade amplitude and duration of saccades [16].

The current study extends the earlier work on the relationship of eye movements and reading disability in two ways. First, two kinds of control groups are included: not only typically developing language learners but also those with another kind of specific learning disability (SLD), namely dysgraphia (impaired handwriting). Although one can have dysgraphia without any reading disability, ability of normally developing readers to write letters has been shown to affect learning to read words [17,18]. Moreover, eye movements are involved as writers read the sentences and text they are writing [19,20]. Thus, it is of interest to know whether eye movements during sentence reading comprehension do or do not differ between those with handwriting disability only and those with reading disability, and if so, how.

Second, eye movements were collected during a brain imaging session while participants were reading sentences. Studies that collect both brain imaging data and cognitive-behavioral data on eye movements may therefore add to understanding of how the brain receives through the eyes information from the external environment about written sentences during reading and integrates that information with internal resources for reading comprehension. On the one hand, cognitive psychology research has a longstanding tradition of collecting behavioral measures to understand how eye movements function during tasks such as sentence reading comprehension. On the other hand, various brain imaging studies have shown that many parts of the brain are involved in eye movements not only for literacy tasks but also for many other kinds of tasks as well: (a) the vestibular system, which is the only sense to operate solely below the cortex, perceives eye movements in mental space; (b) the twelve cranial nerves connected to the central nervous system; (c) the optic nerve which receives visual stimuli from the retina; (d) the abducens that specializes in eye movements; (e) the cortical primary sensory region (BA 1, BA 2, and BA 3) that receives sensory stimulation from movements of the eyes; (f) cortical primary motor region (BA 4) involved in movement of the eyelid and eyeball; (g) the posterior cortical regions in occipital, temporal, and parietal regions involved in processing incoming stimuli during fixations; and (h) the many frontal regionsinvolved in planning and self-regulating eye movements [21]. The goal of the current study was, therefore, to draw on both cognitive behavioral measures and brain measures to study behavioral-brain relationships in the eye movements of developing readers who do and do not have specific learning disabilities (SLDs) in written language—word reading as in the case of dyslexia or handwriting as in the case of dysgraphia.

Toward this goal, following MRI scanning at the beginning of the imaging session, Diffusion Tensor Imaging (DTI) was performed followed by Functional Magnetic Resonance Imaging (fMRI) connectivity scans while participants completed sentence reading comprehension tasks. Because current imaging methods typically do not allow scanning of the retina or optic nerve, the focus of the DTI scanning was on the following bilateral structures beyond the retina and optic nerve: optical radiation, corticospinal tract, inferior longitudinal fasciculus (which shares connectivity with uncinate), superior longitudinal fasciculus (which shares arcuate fasciculus connectivity with both Broca's and Wernicke's area), and cingulum which provides executive coordination of other brain regions. DTI parameters assessed as indicators of white matter integrity in these ten brain regions included (a) fractional anisotropy (FA, an index of the amount of anisotropy which is associated with myelination [22,23]; (b) relative anisotropy (RA, a ratio of strength of connectivity in one direction to strength of connectivity in all directions [24]; (c) axial diffusivity (AD, diffusivity along principal axis of diffusion); (d) radial diffusivity (RD, diffusivity in directions perpendicular to the principal axis of diffusion, which is associated with degree of myelination, number of branching, exiting fibers, and axon diameter [25,26]; and (e) mean diffusivity (MD, a measure of the ability of water to diffuse in any direction).

fMRI functional connectivity scans of gray matter were then obtained while participants performed a sentence reading comprehension task in which the task was to judge whether sentences with and without homonym foils were or were not meaningful. One region of interest as a seed of connectivity was the left occipital temporal cortex known to be involved in transforming input received through the eyes into visible language codes for written word-forms [27,28]. A second region of interest as a seed of connectivity was the left inferior frontal gyrus known to orchestrate the coordination of language processing throughout the brain [29].

Target words in correct sentences were correct word-specific spellings or homonym foils pronounced the same as a word that would make sense in the sentence but was misspelled. Word-specific spellings were of interest because they draw on integration of word spellings (orthographic codes), word pronunciations (phonological codes), and affixes (morphology codes) as well as associated semantic meaning [30,31]. Word-specific spelling tends to be impaired in SLDs affecting both reading and spelling [32]. In addition, behavioral measures of word-specific spellings (choosing the correctly spelled written words among homonym foils) and of silent word reading fluency (detecting word boundaries in letter strings under timed conditions) were obtained to evaluate whether word spelling or word reading achievement might affect performance on the fMRI sentence reading comprehension task. Homographs were not used in which the same spelling is associated with different semantic meanings (like calf for part of leg and calf for an animal) because the goal was to understand how eye movements might be affected when the reader has to integrate orthography, phonology, and morphology with sentence syntax as well as semantics, but not just draw on semantics to comprehend the meaning of the sentence.

To achieve this goal, five behavioral eye movement outcomes were also collected during the just described fMRI sentence reading comprehension task for each target word (correctly spelled or homonym foil) that determined whether the sentence made sense or not: length of initial fixation, dwell time (total fixation time across all fixations), total number of fixations, and total number of two kinds of regressions—regressions in and regressions out. Of interest was how often the groups (with a reading disability or with a handwriting disability or without an SLD in written language) were likely to overshoot the target word during a forward saccade and then have to regress back in to the target word versus likely to regress from the current target word out to a previous fixation on a prior target word. For the first kind of regression, the eyes move in a backward direction from the current fixation beyond the target word to the earlier target word in the sentence. For the second kind of regression, eyes move from the currently fixated word to a previous fixation or region of interest before the target word. Both regressions involve eye movements back. What differs is whether the movement is back from a fixation beyond the current target word to a fixation on that target word, or from a fixation on the current target word to fixation on a prior target word.

To account for more regressions in or more regressions out, if observed in comparisons of diagnostic groups, a silent reading task was given that requires detection of as many word boundaries as possible in rows of letter strings within a 3-minute time limit. Distractibility by other letters in the row before and after the letter series that corresponds to a correctly spelled word can lower the score on the measure. Therefore the results of this test could be used to test Rayner and colleague's hypothesis that in those with dyslexia letters in parafoveal vision may interfere with the processing of a currently fixated word [12]. Silent word reading was measured because at the grade levels studied (fourth through ninth grade), most reading is silent not oral at school.

Although eye movement studies are traditionally conducted with participants sitting in upright position, collecting eye movement data while scanning brains required that participants lie on their backs. There is a limitation to how long participants can lay on their back without movement, which introduces movement artifact. Thus, participants performed tasks during which eye movements are recorded for shorter periods of time during brain scanning than in traditional experiments. This limitation of potential movement artifact is offset by the potential advantages of combining eye tracking with brain scanning to investigate the relationship of eye movements to both the early brain processing after the eyes receive the initial visual messages from the read written sentence in the external environment and during the decision process of reading comprehension.

Altogether four hypotheses were tested. The first two pertained to the eye movements on which the control group without SLD and the control group with dysgraphia were compared, and the group with dyslexia was compared to both control groups. Given the findings of Jainta and Kappoula [13], Kirby and colleagues [14], and Pavlidis [15] that those with dyslexia showed more fixations, longer fixations, and more regressions, the current study focused on these eye movement parameters rather than amplitude. The first tested hypothesis was that the two control groups (controls without SLDs in written language referred hereafter as the non-SLD controls and the dysgraphia group) would not differ significantly from each other on eye movement measures because neither group has problems in silent reading comprehension. The second tested hypothesis was that the dyslexia group would differ from both control groups (non-SLD controls and the dysgraphia group) on the eye movement outcomes. The third tested hypothesis was that differences among these diagnostic groups would be found in DTI white matter integrity indices for structures beyond the optic nerve that may be involved in eye movements; and these DTI integrity differences would be related to the behavioral measure of silent reading fluency, which is more sensitive to written word stimulus input, than to access to and discrimination among word-specific spellings stored in long-term memory relevant to differentiating homonyms and homonym foils. The fourth tested hypothesis was that differences among these diagnostic groups would also be found for fMRI connectivity from a seed (a) in left occipital temporal cortex involved in integrating input from the visual (occipital) cortex with the language (temporal) cortex to create representations of written-word forms; and (b) in left anterior frontal cortex involved in coordinating the word-level and syntax-level processes involved in sentence comprehension.

## Methods

### Participants and Diagnostic Assessment

Procedures used had been approved by the Institutional Review Board where the study was conducted and complied with the ethical guidelines of the American Psychological Association. Twenty nine children who were right-handed and did not wear metal that could not be removed gave assent and their parents gave informed consent for them to participate. Each of the participants, who were in grades 4 to 9 (ages 9 to 14), first completed assessments while their parents completed extensive questionnaires about developmental, medical, family, and educational history and rating scales about current functioning. Based on differential diagnosis guidelines [32], informed by levels of language in the reading, and brain [33], and drawing on over two decades of programmatic research on differential diagnosis of dyslexia and dysgraphia [34,35] assessment results were used to assign participants to a control group without SLDs or one of two diagnostic groups each defined on the basis of a profile (pattern) of scores and educational history rather than a single measure alone with an arbitrary cut-off.

Criteria for **typically developing oral and written language learners profile** (*n*= 10, controls) were as follows: scored at or above - 2/3 SD, the lower limit of the average range, at the 25^th^ %tile or a standard score of 90 on *multiple* normed measures of handwriting, word reading/spelling, and listening and reading comprehension and oral and written expression, had Verbal Reasoning Index, shown in research to be the best predictor of the Wechsler Scale of academic achievement [see 34,35], of at least 80 (-2/3 SD), the lower limit of the low average range, and had no parental reported current or past history of difficulties in any of listening or reading or oral or written language skills.

Criteria for **dysgraphia profile** (*n*=9 were as follows: scored below the lower limit of the average range *on at least two* normed measures of handwriting (subword letter production), showed no evidence of reading disability on normed measures, had verbal reasoning at least within the lower limits of the low average range, and had parent reported current and past history of persisting handwriting problems despite intervention but not of reading problems.

Criteria for **dyslexia profile** (*n*=10) were as follows: scored below the population mean and at least one standard deviation below Verbal Comprehension Index on at least two normed measures of word reading for real and/or nonwords and spelling read and/or nonwords, have parent reported current and past history of persisting word reading and spelling problems despite intervention, and have no history of early emerging problems in understanding or producing oral language before kindergarten or during the school years that fall outside the normal range (e.g., specific language impairment). Research has shown that this profile differentiates between those with reading problems due to dyslexia (impaired word level reading and spelling) rather than developmental disabilities or other neurogenetic or brain conditions, and allows for identification of dyslexia in both individuals whose Verbal Comprehension Index falls at least within the low average range or falls in the gifted range (superior and very superior) in families with multi-generational history of dyslexia [34].

As part of this diagnostic assessment, all participants were given the *Test of Orthographic Competence (TOC)*, which assesses word-specific spelling [36], and the *Test of Silent Word Reading Fluency* [37]. Both of these measures assess at the behavioral level what has also been shown to be hallmark word-level impairment at the brain level in dyslexia [32].

Racial diversity was representative of the relative frequency of racial groups in the area in which the study was conducted (majority European Americans, then Asian Americans, then African American, Native Americans, and Pacific Islanders, as well as mixed races). Parental education ranged from less than high school to beyond college, but the mode was college-level.

### Brain Imaging Procedures

All scans were acquired at the Diagnostic Imaging Sciences Center in collaboration with the Integrated Brain Imaging Center and had Institutional Review Board approval. Each participant was screened for MRI safety before entering the scanner. Physiological monitoring was performed using the Philips pulse oximeter placed on the left hand index finger for cardiac recording; and respiration was recorded using the Philips bellows system where the air-filled bellows pad was placed on the abdomen. Head-immobilization was aided by using the Crania, Elekta inflatable head-stabilization system.

Prior to entering the scanner, students were given sample sentences to read and score as to whether the sentence did or did not make sense. Sentences that did make sense had only correctly spelled words, but sentences that did not make sense had one incorrectly spelled word (homonym foil), which if pronounced sounded like a real word that would make sense in the sentence. To be certain the students understood the task correctly, a score of 90% correct in this training task was required to participate in the scanning. After entering the scanner, students were asked to lie still while the MRI and DTI scans were in progress.

The following MRI series were scanned at the beginning of the session as a reference for the other scanning: **1) 3-plane scout view with gradient echo pulse sequence**: TR/TE 9.8/4.6ms; Field of view 250×250×50mm; acquisition time 30.3s; **2) reference scan (used in parallel imaging) with gradient echo pulse sequence**: TR/TE 4.0/0.75ms; Field of View 530×530×300mm; acquisition time 44.4s; **3) fMRI scan with echo-planar gradient echo pulse sequence (single shot):** TR/TE 2000/25ms; Field of view 240×240×99mm; slice orientation transverse, acquisition voxel size 3.0×3.08×3.0mm; acquisition matrix 80×80×33; slice thickness 3.0, SENSE factor in the AP direction 2.3; epi factor 37; bandwidth in the EPI frequency direction 1933Hz, SoftTone factor 3.5, sound pressure 6.1dB, 5 dummy scans; fold over direction AP, dynamic scans 387, acquisition time 13:08min/s; and **4) diffusion tensor imaging (DTI) with echoplanar spin-echo diffusion pulse sequence:** TR/TE 8593/78ms, slice orientation transverse, Field of view 220×220×128mm, voxel size 2.2×2.2×2.0mm, b values 0 and 1000, output images 1 b value at 0 and 32 b values at 1000 with 32 different diffusion vector non-colinear directions, SoftTone factor 4.0, sound pressure 3.1dB, bandwidth in the EPI frequency direction 1557.7Hz, epi factor 57, acquisition time 9:35.7min/s.

Next DTI scans were collected during which no task was performed. Five DTI parameters of white matter integrity were measured—fractional anisotropy (FA); relative anisotropy (RA); axial diffusivity (AD); radial diffusivity (RD); and mean diffusivity (MD) in ten brain regions— bilateral optical radiation, corticospinal tract, inferior longitudinal fasciculus, superior longitudinal fasciculus, and cingulum.

Then when the fMRI connectivity scanning began, participants were instructed to press the yes box if the sentence could be a real sentence that is meaningful because all the words are spelled correctly and make sense in the sentence, but to press the no box if the sentence is not meaningful because one word does not make sense in the sentence. The “no” items differed from the “yes” items by only one word, which was a homonym foil. This is an example of a no sentence: “The bee, witch buzzes, can sting you.” This is an example of a yes sentence:“ The bee, which buzzes, can sting you.” Correct and incorrect versions of the sentences were presented randomly but occurred equally often. The target homonym could occur in any sentence position except the last. The computer advanced to the next sentence when the button was pushed. This procedure was in effect for a total of 2-minutes in which a participant performed the sentence reading comprehension task multiple times.

The participants' eye movements were recorded during the two minutes of the sentence-reading comprehension task while in a Philips 3T Achieva scanner (release 3.2.2, with a 32 channel head coil). Eyelink Software from SR-Research (version 1.1.1 Ottawa, Canada) was used to analyze eye fixations. Analysis was calibrated by directing the participants to fixate a dot on the screen without moving their heads; the dot moved up/down and right/left, thereby setting the limits for the x- and y-axes of eye motion. Eye-tracking software was used to follow the eye movements of each participant while reading each sentence and record each of these five measures related to fixations (initial fixation time, total fixation/dwell time, and total number) and saccades (regressions in and regressions out).

### Data Analyses

#### Eye movement outcomes

Each eye movement outcome was based on the single interest area representing the target word in the incorrect sentence, or its equivalent interest area in the correct version of the sentence. Between participant ANOVAs were used to evaluate main effects for group for each of the five eye movement dependent measures: First Fixation Duration—the time in milliseconds of the first fixation on the target word; Dwell Time (also referred to as gaze duration)—the total cumulative time in milliseconds that a participant fixated on the target word across all fixations; Total Fixation Count—the cumulative number of fixations on the target word; Regression In — the average number of eye movements from current fixation back to the target word; and Regression Out—the average number of eye movements from currently fixated target word in a backward direction to a previous fixation.

#### DTI parameters

A series of between participant ANOVAs were performed to determine if there were significant group effects (differences among the three diagnostic groups) on each combination of the five parameters for white matter integrity—fractional anisotropy (FA); relative anisotropy (RA); axial diffusivity (AD); radial diffusivity (RD); and mean diffusivity (MD)—and the ten bilateral brain regions beyond the retina and optic nerve—optical radiation, cortico spinal tract, inferior longitudinal fasciculus, superior longitudinal fasciculus, and cingulum which provides executive coordination of other brain regions. For those DTI parameters for a specific brain region showing main effects for group, then posthoc tests compared the dysgraphia and non-SLD control groups, the dyslexia and non-SLD control groups, and the dysgraphia and dyslexia groups. Of interest, were the specific brain region and DTI parameter associated with it on which each of these comparisons between groups showed significant differences. All of the DTI parameter values are multiplied by 10, 000 to help with analysis software, for example an FA value of 5082.32 below in the results can be converted to 0.5082 (original FA values) by dividing by 10,000.

#### fMRI connectivity

A map was generated from the left temporoocipital cortex OTC (MNI -50,-60,-16 mm, between Jülich atlas labels GM_Visual_cortex_V4_L and WM_OptiC_radiation_L) and in the left inferior frontal gyrus, IFG (MNI -52,20 34 mm, Jülich atlas labelGM_Broca's_area_BA44_L). Functional images were corrected for motion using FSL MCFLIRT [35], and then high-pass filtered at sigma = 20.83. Motion scores (as given in the MCFLIRT report) were computed for each participant and average motion score (mean absolute displacement) for each of the groups: control group (*M*= 0.570, *SD*=1.1291), dysgraphia group (*M*=0.245, *SD*=0.307), and dyslexia group (*M*=0.369, *SD*=0.358). None of the motion scores were significantly different from one another. Default parameters in AFNI3s 3dDespike were used to identify and remove Spikes. FSL3s slicetimer was used to apply slice-timing correction and a 3D Gaussian kernel with FWHM = 4.0 mm was used to perform spatial smoothing.

Time series motion parameters and the mean signal for eroded (1 mm in 3D) masks of the lateral ventricles and white matter (derived from running FreeSurfer3s reconall on the T1-weighted image) were analyzed. Co-registration of functional images to the T1 image was performed using boundary based registration based on a white matter segmentation of the T1 image through epi_reg in FSL. The MPRAGE structural scan was segmented using FreeSurfer software; white matter regressors were used to remove unwanted physiological components.

Oxford's fMRIB software library (FSL) randomize, which performs permutations and threshold-free cluster enhancement, was used to control for multiple comparisons. A global design matrix was used as part of the GLM model in software randomise to make the group statistical maps from the 2 seeds for the 3 groups (control, dysgraphia, dyslexia), as described by FSL guidelines in this weblink (http://fsl.fmrib.ox.ac.uk/fsl/fslwiki/glm#Single-Group_Average_.28One-Sample_T-Test.29). fMRI time-series were averaged within regions of interest (ROIs) formed from a 15 mm sphere centered at each seed. The averaged time-series at each ROI was correlated with every voxel throughout the brain to produce functional connectivity correlation maps, converted to z statistics using the Fisher transformation. These group maps show where in the brain there was significant functional connectivity from the seed point to other regions in the brain. Custom software, based on the Jülich histological (cyto-andmyeloarchitectonic) atlas [38,39] and written in Fortran, was used to identify and quantify the brain regions which were significantly connected with each seed point. ANOVAs were used to test for main effects for diagnostic magnitude of fMRI connectivity from the two seed points, and interaction between diagnostic group and two seeds of fMRI connectivity.

#### Relationship of eye movement outcomes, word spelling and word reading achievement, and DTI parameters and fMRI connectivity for diagnostic groups

Correlations were then computed between the brain, the eye movement outcomes that differentiated the two control and dyslexia groups and normed measures of silent word reading and word-specific spelling. Choosing which eye movement measures to include in the correlations was informed by the results of the analyses that identified main effects for diagnostic group differences. Likewise, choosing which seeds and regions of connectivity to include in the correlations was informed by substantial past research on dyslexia [21].

## Results

### Diagnostic Group Differences on Five Eye Movement Outcomes

ANOVAs with diagnostic groups as a between participant variable identified significant main effects for groups on three of the five eye movement outcomes. See Table 1 for means and standard deviations and *F*- values for dwell time (total duration of fixations in seconds), total fixations (total number of fixations), and total number of regressions in during saccades, for all of which the main effect for diagnostic groups was statistically significant. The diagnostic group effects were not significant for initial fixation time or for regressions out during saccades; and so neither of these eye movement outcomes was considered in further analyses. Overall total fixation (dwell) time in seconds during the fMRI silent reading comprehension task was longest for the dyslexic group, next longest for the dysgraphia group, and shortest for the non-SLD control group. Overall the total number of fixations during the fMRI silent reading comprehension task was greater for the dyslexia group and about the same for the dysgraphia group and the non-SLD control group. The average number of regressions in during saccades was higher in the dyslexia group than in the dysgraphia group or non-SLD control group. Additional analyses comparing groups two at a time showed that the dysgraphia group did not differ significantly from the non-SLD control group in total fixation dwell time, total number of fixations, or regression in. However, the dyslexia group differed significantly from the non-SLD control group on total fixation dwell time, total number of fixations, and regressions in. In addition, the dyslexia group and the dysgraphia group differed on total fixation dwell time, total number of fixations, and average number of regressions in. See Table 1.

### Diagnostic Group Differences in fMRI Functional Connectivity

**Figure 1:**
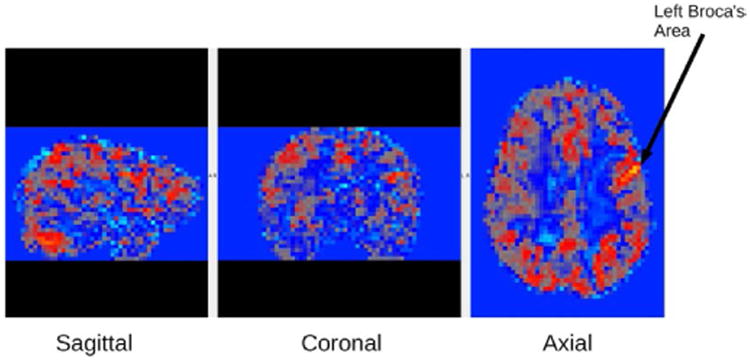
fMRI connectivity example map from one of the participants. This fMRI connectivity map is based on connectivity from the left Broca's seed region shown by the arrow. From left to right Sagittal, Coronal, Axial images are shown for this map.

An example fMRI connectivity map is shown in Figure 1. Mixed ANOVAs with Diagnostic Groups as a between participant variable and brain connectivity networks as a within participant variable were performed for the two networks of interest. These two networks of connectivity were selected based on (a) initial analyses, which controlled for multiple comparisons, and showed they were of statistically significant magnitude; and (b) their theoretical relevance to existing research on reading disability—one related to the regions where incoming visual information is transformed into written words, that is, visible language, and one related to regions involved in the executive functions for the reading comprehension process [21]. These networks were (a) from left occipital temporal cortex (L OTC) with right BA44 in Broca's area; and (b) from left inferior frontal gyrus (L IFG) with right inferior fronto-occipital fasciculus (R IFOF).

Results showed a non-significant main effect for diagnostic groups, *F*(2,26)=1.13, *p*=.34 (eta square .00); a non-significant main effect for brain connectivity networks, *F*(1,26)=1.06, *p*=.31 (eta square .039); and non-significant interaction between seeds and diagnostic groups, *F*(2,26)=.64, *p*=.53 (eta square .047). Although the magnitude of each of these networks from seeds on left—one posterior and one anterior—was statistically significant, connectivity from the seeds was not significantly different across diagnostic groups. Nevertheless, each of the diagnostic groups showed different patterns of significant correlations between magnitude of connectivity from each of these seeds to a specific brain region and the eye movement outcomes and measures of word-specific spelling or silent word reading fluency across the diagnostic groups, which are described next.

### Correlations among Eye Movement Outcomes, fMRI Connectivity, and Word Spelling/Reading

Correlations were then examined between the three eye movement outcomes showing statistically significant main effects for group, significant magnitude of fMRI connectivity between left occipital temporal cortex (L OTC) with right BA 44 in inferior frontal gyrus (L IFG) (a back-front neural pathway) or left inferior frontal gyrus (L IFG) with right inferior fronto-occipital fasciculus (R IFOF) (a front-back neural pathway), and two normed measures of word-specific spelling (TOC) and silent word reading fluency (TOSWRF) relevant to the fMRI task requiring accurate homonym foil detection for correct sentence reading comprehension. For the fMRI reading comprehension task, correlations were analyzed separately for both correct (no homonym foils) and incorrect (homonym foil) items. Results are described first for the profile of each diagnostic group separately and then compared across profiles.

#### Non-SLD control group profile

The following significant correlations involved fMRI connectivity: L OTC seed and L IFG seed, r=.64, p <.05, L IFG seed and regressions in during saccades for correct items r=.74, p <.01, and L IFG and TOC word specific spelling, r=-.80, p<.05. The following significant correlations involved interrelationships among eye movement outcomes: total fixations correct items and total fixations incorrect items, r=.75, p<.01, dwell correct items and dwell incorrect items r=.70, p<.05, dwell correct items and total fixation correct items, r=.87, p<.001, dwell correct items and total fixation incorrect items r=.82, p<.001, dwell incorrect items and total number of fixations incorrect items r=.85, p<.01, regressions in incorrect items and total fixations incorrect items r=.69, p<.05.

#### Dysgraphia group profile

There were no significant correlations involving fMRI connectivity. The following significant correlations among eye movement outcomes were observed: total fixations correct items and total fixations incorrect items r=.76, p<.05, dwell correct items and dwell incorrect items r=.73, p<.05, dwell correct items and total fixations correct items r=.94, p<.001, dwell correct items and total fixations incorrect items, r=.83, p<.01, dwell incorrect items and total fixations incorrect items r =.89, p<.001, total fixations correct and total fixations incorrect, r=.75, p<.05, total fixations incorrect and total dwell correct items r=.83, p<.01, total fixations incorrect items and total dwell time incorrect items r= .89, p<.001, dwell correct items and regressions in incorrect items r=.71, p<.05, and total fixations correct items with regressions in incorrect items r= .86, p<.01. The two measures of word reading and spelling were correlated: TOWSWRF silent word reading fluency and TOC word-specific spelling, r=.75, p<.05. Word-specific spelling was negatively correlated with three eye movement outcomes: dwell correct items and word-specific spelling r= -.68, p<.05, dwell incorrect items, and word-specific spelling, r=-.68, p<.05, and total fixations incorrect items and word-specific spelling r= -.68, p<.05.

#### Dyslexia group profile

The two neural pathways were correlated with each other but not with any eye movement outcomes: L OCT seed and L IFG seed r =.76, p<.01. Two eye movement outcomes for correct items only were correlated with TOC word-specific spelling: dwell time correct items and word specific spelling, r= -.66, p<.05, and total number of fixations correct items and word specific spelling, r= -.66, p<.05.

#### Comparison of profiles

Only the non-SLD and dyslexia groups, not the dysgraphia group, showed correlation with the fMRI connectivity for the two networks included in the correlations. However, only the non-SLD control group, not the dyslexia group, showed a significant correlation between the fMRI connectivity and eye movements (from L IFG seed and regressions in during saccades for correct items) or written words (TOC word-specific spelling).

Only for the non-SLD control and dysgraphia groups were any eye movement outcomes correlated with each other; and the non-SLD and dysgraphia groups shared six of these in common all involving dwell time (total fixation time). In addition, the dysgraphia group, but not the non-SLD control group, had significant correlations between total fixations incorrect and total dwell correct items, total fixations incorrect items and total dwell time incorrect items, dwell correct items and regressions in incorrect items, total fixations incorrect items and total dwell time incorrect items, and total fixations correct items and total regressions in incorrect items.

In contrast to the dysgraphia group that showed significant correlations between total fixations incorrect items and TOC word-specific spelling, the dyslexia group showed significant correlations between total fixations correct items and TOC word-specific spelling and between total fixation time (dwell time) and TOC word-specific spelling.

Clearly, compared to the non-SLD control group, the dysgraphia group showed more correlations with eye movements involving incorrect items. Also, the dysgraphia group showed correlations between TOC word-specific spelling and total fixation incorrect items and between TOC word-specific spelling and dwell time incorrect items; but the dyslexia group showed significant correlations between TOC word-specific spelling and total fixation correct items. It is possible that the greater difficulty the dysgraphia group had than the non-SLD group or the dyslexia group with word-specific spelling related to detecting homonym foils in incorrect sentences may be due, at least in part, to the greater co-occurrence of ADHD in dysgraphia than dyslexia [40].

### Selective Attention Dyslexia as Explanation of Eye Movement Outcomes

ANOVA with diagnostic group as a between participant variable showed a significant main effect for TOSWRF silent word reading in a string of letters, a task susceptible to distractibility from other letters on either side of the embedded word, F(2, 28)=4.94, p=.015. TOSWRF scores were considerably higher in the non-SLD control group (M=105.20, SD=11.40) and dysgraphia group (M=105.44, SD=18.05) than in the dyslexia group (M=89.10, SD=9.18).

### Diagnostic Group Differences in DTI Indicators of White Matter Integrity

**Figure 2:**
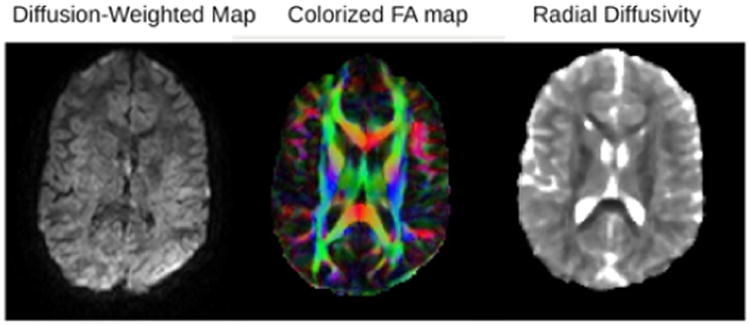
Example DTI images from one participant. From left to right: Example of diffusion-weighted image, fractional anisotropy map and radial diffusion map. The color in the FA map is based on the fiber direction

**Figure 3:**
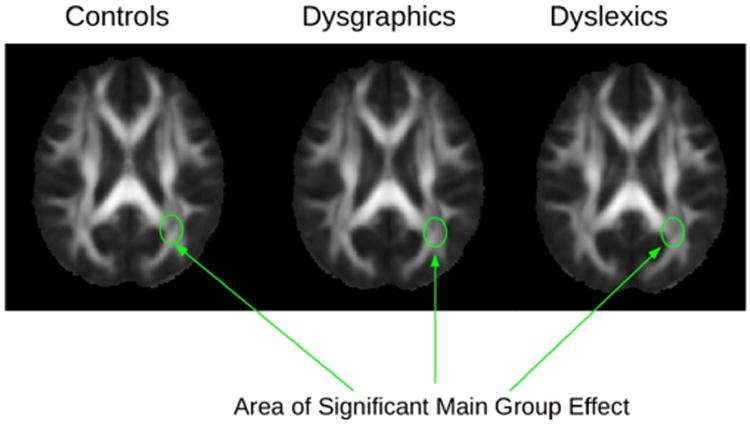
DTI mean fractional anisotropy (FA) maps from the Control group, the Dysgraphic group, and the Dyslexic group. The arrows show the location in the brain (left optic tract) for the main statistical effect for diagnostic group for FA.

Example diffusion and DTI maps for one subject are shown in Figure 2. A significant main effect for diagnostic group was found for the following DTI parameters in these brain regions: fractional anistrophy (FA) in left optic radiation, F(2,26)=3.56, p=.04; radial diffusion (RD) in left corticospinal, F(2, 26)=3.32, p=.05; and radial diffusion (RD) in left superior longitudinal fasciculus, F(2,26)=24.29, p=.03. Figure 3 shows the location of the FA main effect in the left optic radiation which is indicated on each of the diagnostic group mean FA maps. Additional analyses comparing two groups at a time showed that the dysgraphia group did not differ from the non-SLD control group in fractional anistrophy (FA) in the left optic radiation. The dysgraphia group did differ from the non-SLD control group in radial diffusivity (RD) in left corticospinal, F(1,17)=4.47, p=.05 (control, M=4.48, SD=.32; dysgraphia, M=4.88, SD=.52 in units of 10–4 mm^2^/sec), and in left superior longitudinal fasciculus, F(1,17)=8.39, p=.01 (control, M=5.46, SD=.45; dysgraphia, M=6.03 SD=.41 in units of 10–4 mm^2^/sec). The dysgraphia group had a higher value of RD in left corticospinal and in left superior longitudinal fasciculus than did the control group. Overall, the dysgraphia group differed from the non-SLD control group in two of the three DTI-brain region combinations on which there was a main effect for group. One of these regions (left cortical spinal) connects hand with peripheral nervous system for the act of writing; the other region (left superior longitudinal fasciculus) connects visual input with visible language processing in cortex. The dysgraphia group did not differ from the non-SLD control in the brain region that is closer to eye, which provides the visual stimulus input for reading.

The dyslexia group differed from the non-SLD control group on fractional anistrophy (FA) in left optic radiation, F(1,18) = 4.65, p=.05 (control, M=0.5082, SD=0.467; dyslexia, M=0.4562, SD=0.502). For FA, the dyslexia group had a lower value than the control group. Overall, the dyslexia group differed from the non-SLD control group on one DTI-brain region combination for which there was a main effect for group— one that is closer to the retina and optic nerve in the transmission of the visual signal. The dyslexia group differed from the dysgraphia group in fractional anistrophy (FA) in left optic radiation, F(1,17)=4.25, p=.05, which provides early visual input during reading from optic nerve and retina. The dysgraphia group (M=0.5006, SD=0.240) had higher FA values than the dyslexia group (M=0.4562, SD=0.602). The difference between the dyslexia and dysgraphia groups was marginally significant (p=.06) on RD in left corticospinal, but not significant on RD in left superior longitudinal fasciculus. Of note, none of the significant DTI findings involved right regions even though the five DTI analyses were performed bilaterally.

## Discussion

### Tested Hypotheses

*The first tested hypothesis* that the two control groups—non-SLD control and non-reading disabled dysgraphia group— would not differ significantly from each other on sentence reading comprehension was supported when the three eye movement outcomes that differentiated diagnostic groups were considered: total dwell time during fixations, total number of fixations, and number of regressions in during saccades. However, when the correlations between eye movement outcomes and magnitude of functional connectivity from a seed in the posterior region and a seed in the anterior region of the brain and achievement in word specific spelling or silent word reading fluency were evaluated, this hypothesis was not supported. Differences between the two control groups in patterns of correlations were observed.

*The second tested hypothesis* that the dyslexia group would differ from the dysgraphia group on sentence reading comprehension was supported for (a) eye movement outcomes that differentiated diagnostic groups—total fixation (dwell) time, total number of fixations, and number of regressions in during saccade, (b) correlations between eye movements with word specific spelling and silent word reading fluency, and (c) connectivity from the left OTC and the left IFG seeds. That is, multiple kinds of differences were observed between two SLD groups that differed in the level of language impairment—subword handwriting or word spelling and reading.

*The third tested hypothesis* that each of two SLD groups would differ from the non-SLD control group on DTI parameters for structures beyond the optic nerve was supported, but with qualifications. The sample as a whole showed a main effect for diagnostic group in fractional anisotropy (FA) in left optic radiation, and in radial diffusivity (RD) in left corticospinal and in left superior longitudinal fasciculus. When the three diagnostic groups were compared two at a time on these DTI indicators of white matter integrity, how each SLD group differed from the non-SLD group and from each other was not exactly the same. The dysgraphia group did not differ from the non-SLD control group on fractional anisotropy (FA) in left optic radiation, but the dyslexia group did; and the dyslexia and dysgraphia group differed from each other on FA in left optic radiation. The left optic radiation is the nearest brain region, which could be scanned, to the retina and optic nerve that transmits the incoming visual signal from the written sentence being read to the cortex Thus, the dyslexia group showed a white matter integrity difference from both control groups—one without an SLD in reading or handwriting and one with an SLD in handwriting but not reading--early in the visual processing during reading that could affect silent reading comprehension of written sentences when words have to be processed in sentence context and eye movements are required to manage this process. At the same time, the dysgraphia and non-SLD control groups differed in radial diffusivity (RD) in left corticospinal and in left superior longitudinal fasciculus, which may be related to the writing problems in dysgraphia. Thus, the nature of the white matter integrity difference appears to be related to nature of the SLD as was the case for the second tested hypothesis.

*The fourth tested hypothesis* was that each of the two SLD groups studied—dysgraphia and dyslexia— would differ from the non-SLD control group on fMRI connectivity from a seed (a) in left posterior cortex involved in integrating input from the visual (occipital) cortex with word-specific language processing in the language (temporal) cortex; and (b) a seed in left anterior frontal cortex involved in coordinating the word-level and syntax-level processes involved in sentence comprehension. Although there was not a significant main effect for diagnostic group for the connectivity from these seeds, the diagnostic groups showed different patterns of significant correlations between magnitude of bilateral connectivity from left occipital temporal cortex with right BA 44 in Broca's area and from left inferior frontal gyrus with right inferior frontal occipital fasciculum and normed measures of word specific spelling and silent word reading fluency.

### Contributions of Current Research on Eye Movements, Brain, and Word Spelling and Reading

Two kinds of prior research findings replicated and were extended. First, prior differences identified between a non-SLD control group and dysgraphia or dyslexia groups on fMRI connectivity on writing tasks and DTI parameters [41] were extended to eye movement outcomes on an fMRI sentence reading comprehension task and DTI parameters. Second, prior research findings showing that those with and without reading disability differ in total number of fixations, duration of fixations, and number of regressions (for regressions in) [13-15] replicated; and these findings were extended to regressions in during saccades for the dysgraphia versus dyslexia comparison.

Furthermore, findings suggest that different aspects of attention are involved in the reading comprehension of those with dysgraphia versus dyslexia. Those with dysgraphia, who are more likely to have co-occurring ADHD [42], may struggle in attending to all the words in sentence syntax relevant to detecting homonym foils that do not fit the meaning of syntax context, thus explaining their poorer performance on sentences with homonym foils (incorrect items). Those with dyslexia, who are more likely to have inattention and difficulty with switching attention [34], may be vulnerable to distractibility from other words during saccades, resulting in more regressions in, or from other letters in the normed measure of silent word reading fluency used.

Three kinds of findings provided converging evidence for Rayner and colleague's selective attention dyslexia hypothesis [43], even though the orthographic confusion task used in many eye movement studies of dyslexia was not used in the current study. Rather the sentence reading task used required the integration of orthographic and other language codes within the context of other written words to detect homonym foils that did not fit the meaning of sentence context. First, the dyslexia group was shown to have a significantly lower score than both control groups on the silent reading fluency measure in which a correctly spelled word embedded in letter strings had to be detected; those with dyslexia may have been distracted by letters on either side of a real word boundary as their eyes moved along the rows of letters. Second, only the dyslexia group made significantly more regressions in during saccades; distractibility from other nearby letters in the sentences during the eye movements may have caused those with dyslexia to overshoot the next word to be fixated, thus necessitating a regression back to fixate on the relevant word. Third, only the dyslexia group exhibited the DTI difference in left optic radiation which may affect the quality of the visible language stimulus that arrives in the cortex for processing of the written words in sentence context. Differentiating homonyms and homonym foils, both of which could be a correct word spelling in some sentence contexts, requires judgement about whether the target word is a correctly spelled word for the meaning of the sentence syntactic context in which it appears.

Thus, the fMRI results are also consistent with Rayner's conclusion that problems in eye movements are the result not cause of reading disability [43]. As the comparisons of the profiles for the different diagnostic groups showed, for the dyslexia group, word-specific spelling knowledge is negatively correlated with dwell time and total number of fixations during eye movements. Lower word-specific spelling knowledge is related to longer dwell time and more fixations; and higher word-specific spelling knowledge is related to shorter dwell time and fewer fixations. That is, not just incoming visual stimuli (written words) via left radiation but also existing representations in memory, as assessed by word-specific spelling knowledge, contribute to eye movements during silent sentence reading comprehension and when less developed may interfere with eye movements. Processing word-specific spelling requires integration of orthographic knowledge of letter identity, position, and sequence, phonological knowledge of corresponding sound identity, position, and sequence, morphological knowledge of bases and their transformation via affixes, and semantic meaning (vocabulary) [32,34,35]. Although across diagnostic groups, the cortical functional connectivity was of comparable magnitude for each of these networks, functional fMRI connectivity from L OCT with R BA44, along a back-front axis, and from L IFG with R I FOF, along a front-back axis, exhibited different patterns of correlations for the diagnostic groups with eye movement outcomes for sentence reading comprehension during the fMRI scanning and with measures of word-specific spelling or silent word reading fluency.

### Limitations, Future Research Directions, and Conclusions

The current cross-disciplinary study was designed to replicate findings of the well-established field of eye movement research in individuals without and with reading disabilities such as dyslexia and also extend that research to another SLD involving handwriting rather than reading. Thus, the research was both confirmatory and exploratory [44]. Exploratory studies such as the brain bases for eye movements during sentence reading comprehension, which are costly and conducted with relatively small samples and have related power issues [45], will require additional future research to evaluate replicability with other samples and even reading comprehension tasks.

Indeed further research is needed on the impact of anomalies in transmission of information from left radiation on the binocular coordination across visual fields and sides of the brain [46] in individuals with dyslexia compared to other clinical groups and controls and clarification of how such transmission anomalies are probably not the same as visual perception dysfunctions requiring visual training apart from language instruction to overcome. Although there are other studies of developmental dysgraphia (struggle to acquire handwriting) or acquired dysgraphia (loss of the previously acquired handwriting skill), relatively few studies have investigated the relationships between eye movements and struggle to learn handwriting or loss of handwriting. The current study did not find differences between the dysgraphia group and the non-SLD control group on total fixation duration, total number of fixations, or total number of regressions in during saccades, but it did identify two differences between the dysgraphia group and non-SLD control group in DTI indicators of white matter integrity in brain regions that may receive visible language input from the eyes and have connectivity with hands for output of visible language through the motor system: in radial diffusivity (RD) in left corticospinal and in left superior longitudinal fasciculus. These white matter anomalies may be related to the writing problems in dysgraphia shown to involve both orthographic coding in the “mind's eye” and planning sequential finger movements [34]. These initial observations warrant further investigation in other samples to evaluate if they replicate and to extend them in new research designs such as the emerging line of research on both eye movements and finger movements [42].

A word of caution is in order regarding the treatment significance of the current findings. These results should not be interpreted as evidence that students with dyslexia need visual training apart from explicit instruction in reading and spelling written words, that is, visible language. A recent study showed that for students with dyslexia of comparable age as in the current study, computer-presentation of text to be read so that each sentence is presented one word at a time (eliminating the distractibility from other words in a sentence) resulted in better reading comprehension than presenting each sentence one added word at a time (allowing for the possibility of regressions out due to distractibility from other visually displayed words (authors, manuscript in preparation). Also the DTI results of the current study show the relationship of language by hand (writing) and language by eye (reading) for the dysgraphia group, consistent with much cognitive research showing a writing-reading relationship, including the benefits of note taking in handwriting for reading comprehension [47].

Finally, future research might also include students who meet research criteria for impaired morphological and syntactic impairment (specific language impairment, SLI, or oral and written language learning disability, OWL LD). Students with SLI/OWL LD might be compared to the same diagnostic groups as in the current study but on a reading comprehension task that does and does not have affix foils for suffixes that mark part of speech in sentence syntax context.

To summarize, both bottom-up (left radiation FA) and top-down (left corticospinal RD) [7] white matter pathways are involved in sentence reading comprehension. For those with dyslexia, white matter integrity in the left optic radiation prior to cortex, may interfere with the quality of incoming written words in sentence units transmitted along the bottom-up axis receiving information from the external world; and gray matter functional connectivity along back-front (from left to right) and front-back (from left to right) axes contributes to processing the received information via access to existing mental representations of written words, which may vary in quality and ability to overcome poor quality input. The important point is sentence reading comprehension is not dependent solely on the sensory input or processing a single word apart from the context of the multi-word syntax in which it occurs. Consistent with orchestration of mind theory [44] eye movements, which play a role in processing specific words within the context of multiple words, travel across space and time in the inner internal mental universe housed in the brain to process both single words during fixation and all the words in a sentence across saccades. To do so, they draw on both minds in motion and input from the external world to translate visible language into cognitions.

In conclusion, eye movements play a role in the executive coordination of the mental world. Sentence reading comprehension cannot be understood as purely a visual versus a language process but rather involves the integration of visual and language processes. Although at one time dyslexia was thought to be solely a visual perception deficit, after several decades of research, numerous findings have provided converging evidence for the role of language in the etiology of dyslexia. Integrating eye movement and brain research in a systems approach has an important contribution to make in advancing knowledge of how the brain that processes incoming information from the external world through the eyes and then via eye movements navigates the internal mental world to transform, via access to existing mental representations and operations, the visual input into mental representations of visible language and related cognitions during reading comprehension.

## Figures and Tables

**Table 1:** Eye Movement Outcomes^a^ for which Main Effects for Diagnostic Groups Were Statistically Significant and Follow-Up Comparisons of Two Groups at a Time.

Group	Fixation Time		Fixations		Regressions In		df	F	p
	M	SD	M	SD	M	SD			
1 Non-SLD	761.21	213.23	3.00	.93	.62	.13			
2 Dysgraphia	871.58	358.18	2.91	.74	.56	.13			
3 Dyslexia	1,486.13	681.28	4.65	1.63	.73	.09			
	Main						(2,26)	7.00	.004
			Main				(2, 26)	6.77	.004
					Main		(2, 26)	4.96	.015
1 *vs.* 2	Comparison						(1,17)	.68	.42
1 *vs.* 3	Comparison						(1,18)	10.30	.005
2 *vs*. 3	Comparison						(1,17)	5.84	.027
1 *vs.* 2			Comparison				(1,17)	.06	.80
1 *vs.* 3			Comparison				(1,18)	7.69	.01
2 *vs.* 3			Comparison				(1,17)	8.62	.009
1 *vs.* 2					Comparison		(1,17)	1.15	.30
1 *vs.* 3					Comparison		(1, 19)	4.24	.05
2 *vs.* 3					Comparison		(1,17)	.004	10.99

a All eye movement outcomes are totals
